# Construction and validation of an algorithm for disinfection of ambulances transporting patients with contagious infectious diseases

**DOI:** 10.1590/0034-7167-2022-0081

**Published:** 2022-11-28

**Authors:** Geraldo Magela Salomé

**Affiliations:** I Universidade do Vale do Sapucaí. Pouso Alegre, Minas Gerais, Brazil

**Keywords:** Personal Protective Equipment, Occupational Health, Occupational Exposure, House Calls, Contagious Infectious Diseases, Equipo de Protección Personal, Salud Laboral, Exposición Profesional, Visita Domiciliaria, Enfermedades Transmisibles, Equipamento de Proteção Individual, Saúde do Trabalhador, Exposição Ocupacional, Visita Domiciliar, Doenças Infectocontagiosas

## Abstract

**Objectives:**

to develop and validate an algorithm to guide professionals in cleaning and disinfecting ambulances after transferring patients with contagious infectious diseases.

**Methods:**

the study was conducted between September and November 2021. The developed algorithm was validated by 104 judges, including nurses, physical therapists, and physicians who care for patients with contagious infectious diseases. It used the Delphi technique and content validity index.

**Results:**

in the first evaluation, the judges considered the algorithm “unsuitable” and “fully suitable”. The algorithm reviewed according to the judges’ suggestions was rated between “suitable” and “fully suitable” in the second evaluation. The overall content validity index was 0.960 and 0.998 in the first and second evaluations.

**Conclusions:**

the algorithm to guide the cleaning and disinfection of ambulances after transferring patients with contagious infectious diseases was developed and validated by specialists in the field, with consensus among the judges in the second evaluation.

## RESUMO

**Objetivos:**

desenvolver e validar um algoritmo para orientar profissionais na limpeza e desinfecção de ambulância após transferência de paciente com doenças infectocontagiosas.

**Métodos:**

estudo realizado entre setembro e novembro de 2021. O algoritmo desenvolvido foi validado por 104 juízes, incluindo enfermeiros, fisioterapeutas e médicos que prestam assistência aos pacientes com doenças infectocontagiosas. Utilizaram-se a técnica Delphi e o índice de validade de conteúdo.

**Resultados:**

na primeira avaliação, o algoritmo foi considerado pelos juízes entre “inadequado” e “totalmente adequado”. O algoritmo revisado segundo as sugestões dos juízes foi classificado entre “adequado” e “totalmente adequado” na segunda avaliação. O índice de validade de conteúdo geral foi de 0,960 e 0,998 na primeira e segunda avaliação, respectivamente.

**Conclusões:**

o algoritmo para orientar na limpeza e desinfecção de ambulância após transferência de paciente com doenças infectocontagiosas foi construído e validado por especialistas na área, havendo consenso entre os juízes na segunda avaliação.

## RESUMEN

**Objetivos:**

desarrollar y validar algoritmo para orientar profesionales en la limpieza y desinfección de ambulancia tras transferencia de paciente con enfermedades transmisibles.

**Métodos:**

estudio realizado entre septiembre y noviembre de 2021. El algoritmo desarrollado fue validado por 104 jueces, incluyendo enfermeros, fisioterapeutas y médicos que asisten a pacientes con enfermedades transmisibles. Utilizada técnica Delphi e índice de validez de contenido.

**Resultados:**

en la primera evaluación, los jueces consideraron el algoritmo entre “inadecuado” y “totalmente adecuado”. El algoritmo revisado conforme sugestiones de los jueces fue clasificado entre “adecuado” y “totalmente adecuado” en la segunda evaluación. El índice de validez de contenido general fue de 0,960 y 0,998 en la primera y segunda evaluación, respectivamente.

**Conclusiones:**

el algoritmo para orientar en la limpieza y desinfección de ambulancia tras transferencia de paciente con enfermedades transmisibles fue construido y validado por especialistas en el área, habiendo consenso entre los jueces en la segunda evaluación.

## INTRODUCTION

Professionals of patient transport services are exposed daily to occupational risks caused by occupational factors at different times during the assistance^(1-2)^. Those professionals are highly likely to contract the disease with the emergence of contagious infectious diseases. It is necessary to develop protocols with preventive measures for occupational hazards to promote more safety for those who perform this type of assistance. Those protocols should include the patient transport technique, the ambulance cleaning and disinfection technique, and the cleaning technique for the equipment inside the ambulance^(3-5)^.

Contagious infectious diseases are those caused by biological agents such as viruses, bacteria, or parasites, and they are transmissible through direct or indirect contact with infected individuals^(3-4)^.

Biological agents such as viruses, bacteria, or parasites remain on equipment surfaces for hours or days and in aerosols and dust for up to three hours^(6)^. Therefore, after transporting patients with contagious infectious diseases in mobile units, the equipment, and its physical structure must be rigorously cleaned and disinfected^(6)^. Those procedures, when performed in the ambulance, substantially reduce the level and frequency of contamination by a biological agent, so it is necessary to train professionals to perform them correctly and effectively systematically^(7)^.

Cleaning the mobile unit can positively and negatively impact the health of the worker and the patient depending on the execution. Incorrect execution of the technique can leave professionals and patients exposed to agents harmful to health. We used algorithms, brochures, booklets, and applications to minimize errors in the cleaning process with the descriptions of the cleaning and disinfection techniques, as well as measures to verify the stages to complete it^(8)^.

Algorithms provide professionals with increased safety and decreased risk of accidents, preventing contamination. They should be elaborated scientifically by reviewing the literature^(9-10)^. Those developed in the health area should be simple, objective, and easily accessible instruments; furthermore, they should offer a complete picture of the care process, serving as a basis for decision-making^(11)^.

This study is part of a development project of educational technology for professionals working in ambulances, providing information about the cleaning and disinfection of the mobile unit after transporting the patient to prevent the spread of contagious infectious diseases to professionals and patients. Thus, by using the algorithms, the professional will help free of harm, safely, and with as little risk as possible.

## OBJECTIVES

To develop and validate an algorithm to guide professionals in cleaning and disinfecting an ambulance after transferring a patient with contagious infectious diseases.

## METHODS

### Ethical aspects

The study complied with Resolution 466/12. It was approved by the Research Ethics Committee of the College of Health Sciences (*Faculdade de Ciências da Saúde*) Dr. José Antônio Garcia at the University of Vale do Sapucaí, located in the city of Pouso Alegre, state of Minas Gerais, Brazil. All participants signed the Informed Consent Form before their inclusion in the study.

### Design, period, and place of study

This study is descriptive, methodological, qualitative, and quantitative, following the SRQR guidelines, developed at General Hospital Samuel Libânio from September to November 2021.

The study consisted of developing and validating an algorithm to guide professionals of patient transport services to clean and disinfect the ambulance after attending to patients with contagious infectious diseases.

### Population; criteria of inclusion and exclusion

The Brazilian standard ABNT ISO/IEC 25062:2014 validated the algorithm recommending a minimum sampling of ten participants for each professional.

A panel of judges was composed of professionals working on the frontline of the fight against contagious infectious diseases. The judges were selected through snowball sampling: after identifying a subject that fit the study’s inclusion criteria, researchers asked that professional to indicate other possible participants.

The inclusion criteria for the judges were: obtained a degree in Nursing, Medicine, or Physiotherapy and working on the frontline of care for patients with contagious infectious diseases. Professionals who agreed to participate in the study but failed to answer or did not submit the questionnaire 15 days after receiving it were excluded from the study.

### Study protocol

The study was developed in two stages: development and validation of the algorithm.

### First stage: algorithm development

A literature review was conducted in the MEDLINE, SciELO, Cochrane, and LILACS databases, using the Health Science Descriptors “disinfection,” “ambulances,” and “personal protective equipment (PPE)” in different combinations with the use of the Boolean operator OR, in Portuguese, Spanish, and English.

For selecting the publications identified in the literature search, we adopted the following inclusion criteria: only primary studies with a direct connection to the subject and original articles published between 2016 and 2021.

We excluded theses, dissertations, monographs, technical reports, and articles that, after reading the abstract, did not match the proposed study object and publications repeated in the databases. The reading of the titles and abstracts was carried out independently by two researchers. In case of doubt about the content of a publication, we include it initially and decide on its selection only after reading the entire text.

The algorithm was built by evaluating the works selected in the literature survey and comprised a sequence of procedures described in six topics: 1) Sequence of donning PPE - This topic indicated the types of PPE and the sequence of donning during the cleaning and disinfection of the ambulance 2) Materials used for cleaning and disinfection of the ambulance - Here, the description of types of materials for cleaning and disinfection of the ambulance after the transfer of a patient with contagious infectious diseases; 3) Disinfection of the driver’s cabin - This topic presents the types of solutions and the technique for disinfecting the driver’s cabin after transferring a patient with contagious infectious diseases; 4) Disinfection of equipment and materials in the ambulance - Description of the types of solutions and the sequence of disinfection of equipment or materials; 5) Cleaning and washing of the ambulance - Description of the technique for cleaning and washing the mobile care unit and the types of solutions to be used.; and 6) Sequence of doffing personal protective equipment - The sequence of doffing PPE must be performed after the presentation of the cleaning and disinfection of the ambulance.

### Second stage: Algorithm Validation

The algorithm developed was validated using the Delphi technique, which uses questionnaires to evaluate the instrument’s content by a panel of judges, seeking a level of agreement of 50% to 100% among them^(12)^. In this study, the algorithm was validated when it reached a 90% consensus among the judges^(12)^.

The evaluation and validation of the algorithm content was conducted by a panel composed of nurses, physicians, and physical therapists working on the frontline of care for patients with contagious infectious diseases.

Each participant in the study received an invitation letter by e-mail that included an initial presentation by the researcher; explanations about the research subject; a copy of the Research Ethics Committee opinion; an Informed Consent Form; a copy of the developed algorithm; an evaluation questionnaire; explanations about the importance of the evaluator for the study and about the evaluation cycles; and instructions to carry out the evaluation and to submit the completed questionnaire within 15 days from the date of the e-mail.

The questionnaire was divided into two parts: four questions to identify the evaluators, including the type of degree, time since graduation, time working in the field and educational background; and evaluation of the algorithm with 13 questions related to clarity, theoretical relevance, practical relevance, as well as types of materials and correct technique to perform the cleaning and disinfection of the ambulance.

Responses to the evaluation questions were arranged on a four-point Likert scale, with “suitable,” “partially suitable,” “ totally suitable,” and “unsuitable” as response options, with instructions for optional descriptive responses. Responses marked by the judges as “suitable” or “totally suitable” were counted. Items rated as “unsuitable “ or “partially suitable” were reviewed based on the suggestions made by the judges and presented in a new round of evaluation, according to the Delphi technique^(12)^.

### Analysis of results and statistics

Absolute and relative frequency was used to present the judge’s evaluation of the algorithm’s content according to the Delphi technique.

The content validity index (CVI) measured the proportion or percentage of judges who agreed with certain aspects of the instrument’s content. The CVI was calculated using the judges’ average number of “suitable” and “totally suitable” responses. The judge’s level of agreement greater than 0.8 (80%) verified the instrument’s content validity^(12)^.

## RESULTS

**Figure 1:**
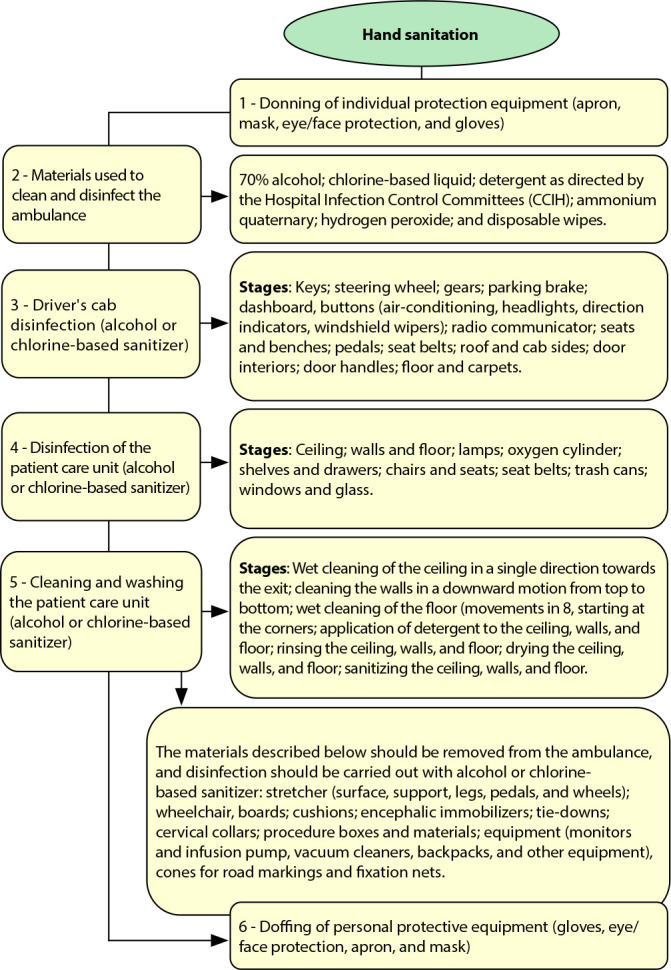
Algorithm to guide health professionals in cleaning and disinfecting the ambulance after attending patients with contagious infectious diseases

A total of 9,322 articles were identified by searching the health sciences databases, from which 2,563 were excluded for being duplicates. The title from 6,759 articles and the abstract from 6,522 were read, resulting in 237 articles to read in their entirety. Of these, 221 were excluded, leaving 16 articles as the theoretical basis for constructing the algorithm (Figure 1).

One hundred four judges validated the developed algorithm. Among the participants, 48 (46.15%) were nurses, 26 (25%) were physicians, and 30 (28.85%) were physical therapists. There were 49 (47.12%) specialists, 29 (27.88%) masters, and 26 (25%) PhDs. Twenty-seven participants (25.96%) obtained their degree within two to five years; Twenty-eight (26%) between five to ten years; and forty-nine (47.12%), more than ten years.

In the first algorithm evaluation using the Delphi technique, the judges rated its content between “suitable” and “ totally suitable.” (Table 1).

**Table 1:** First evaluation of the algorithm content by the judges using the Delphi technique

Questions	Unsuitable		Partially suitable		Suitable		Fully suitable	
(N = 104)	**n**	**%**	**n**	**%**	**n**	**%**	**n**	**%**
Graphic presentation	10	9.62	1	0.96	41	39.42	52	50.00
Easy to read	2	1.92	8	7.69	43	41.35	51	49.04
Sequence of the algorithm	2	1.92	2	1.92	55	52.88	45	43.27
Vocabulary	2	1.92	1	0.96	46	44.23	55	52.88
Content proper to the target audience	1	0.96	1	0.96	50	48.08	52	50.00
Content offers relevant information	0	0	0	0	41	39.42	63	60.58
The text sequence is logical and coherent	2	1.92	8	7.69	43	41.35	51	49.04
The verbal language is easy to understand	1	0.96	0	0	43	41.35	60	57.69
Types of PPE for cleaning and disinfecting the ambulance	3	2.88	2	1.92	49	47.12	50	48.08
Sequence of donning and doffing for cleaning or disinfecting the ambulance	1	0.96	2	1.92	37	35.58	64	61.54
Materials and solutions for cleaning and disinfecting the ambulance	1	0.96	1	0.96	50	48.08	52	50.00
Description of the disinfection technique for the driver's cab	2	1.92	0	0	42	40.38	60	57.69
Description of the ambulance cleaning and washing technique	0	0	0	0	48	46.15	56	53.85

*N - total sample size; n - population size; PPE - personal protective equipment.*

After corrections to the algorithm content, it was reevaluated by the judges. The questions “graphic presentation” and “ easy to read” obtained 99% of agreement among the judges, being considered between “partially suitable” and “totally suitable,” and the other questions were evaluated between “ suitable “ and “totally suitable” (Table 2).

**Table 2:** Second evaluation of the algorithm content by the judges using the Delphi technique

Questions	Unsuitable		Partially suitable		Suitable		Fully suitable	
(N = 104)	**n**	**%**	**n**	**%**	**n**	**%**	**n**	**%**
Graphic presentation	0	0	1	0.96	51	49.04	52	50.00
Easy to read	0	0	1	0.96	47	45.19	56	53.85
Sequence of the algorithm	0	0	0	0	43	41.35	61	58.65
Vocabulary	0	0	0	0	48	46.15	56	53.85
Content proper to the target audience	0	0	0	0	51	49.04	53	50.96
Content offers relevant information	0	0	0	0	41	39.42	63	60.58
The text sequence is logical and coherent	0	0	0	0	48	46.15	56	53.85
The verbal language is easy to understand	0	0	0	0	43	41.35	61	58.65
Types of PPE for cleaning and disinfecting the ambulance	0	0	0	0	49	47.12	55	52.88
Sequence of donning and doffing for cleaning or disinfecting the ambulance	0	0	0	0	38	36.54	66	63.46
Materials and solutions for cleaning and disinfecting the ambulance	0	0	0	0	43	41.35	61	58.65
Description of the disinfection technique for the driver's cab	0	0	0	0	50	48.08	54	51.92
Description of the ambulance cleaning and washing technique	0	0	0	0	48	46.15	56	53.85

*N - total sample size; n - population size; PPE - personal protective equipment.*

Table 3 shows an overall CVI of 0.960 in the first evaluation, corresponding to an agreement of 96% among the judges. In the second evaluation, the CVI was 0.998, indicating a 99.8% consensus of the panel of judges and validation of the instrument.

**Table 3:** Content validity index for the first and second evaluation of the algorithm

Questions	CVI	
	First evaluation	Second evaluation
Graphic presentation	0.894	0.990
Easy to read	0.904	0.990
Sequence of the algorithm	0.962	1.00
Sequence of the algorithm	0.971	1.00
Content proper to the target audience	0.981	1.00
Content offers relevant information	1.00	1.00
The text sequence is logical and coherent	0.904	1.00
The text sequence is logical and coherent	0.990	1.00
Types of PPE for cleaning and disinfecting the ambulance	0.952	1.00
Sequence of donning and doffing for cleaning or disinfecting the ambulance	0.971	1.00
Materials and solutions for cleaning and disinfecting the ambulance	0.981	1.00
Description of the disinfection technique for the driver's cab	0.981	1.00
Description of the ambulance cleaning and washing technique	1.00	1.00
General CVI	0.960	0.998

*CVI - Content Validity Index; PPE - personal protective equipment.*

## DISCUSSION

Some studies point out that the main objectives of educational materials are to inform, increase knowledge, develop, and improve skills, and support decision-making^(13-15)^. The algorithm developed and validated in this study aims to provide safe assistance to professionals who work in transporting patients with contagious infectious diseases and to the individuals transported in the ambulance. This instrument can also help healthcare professionals who transport individuals with contagious infectious diseases, offering procedures for disinfection and cleaning the mobile unit to prevent professionals and users from becoming contaminated during transport^(12-15)^.

The algorithm was developed after a literature review according to several studies that recommend that those instruments should be designed after a literature review and be content-validated^(13-16)^. Thus, the educational technology was created based on scientific subsidies, enabling its implementation in clinical practice, and providing systematized, individualized, and personalized care with less risk to the patient and minimizing damage and adverse events^(13-16)^.

In the first evaluation of the algorithm using the Delphi technique, the judges requested changes in graphic presentation, and the text should be easier to read. After corrections, the algorithm was reevaluated, obtaining a consensus above 99% among the judges, characterizing the content as transparent and objective.

Previous studies that validated the content of educational technology through the Delphi technique concluded that the evaluators’ suggestions should be considered and incorporated into the instrument^(17-18)^. This procedure contributes to better effectiveness of the instrument and implementation of the material in healthcare and/or educational institutions, allowing the target audience to understand the content of the material and be encouraged to use it^(17-18)^.

When an algorithm is validated by professionals with experience in the field and its CVI is above 0.90, the evaluators consider the algorithm’s content relevant. That is essential for using the instrument in clinical practice and health education. The scientific validation by the target audience provides credibility to the algorithm^(3,8,14,19)^.

The algorithm developed in this study benefits the professionals because specialists in the field and experience in transporting patients with contagious infectious diseases validated it. This instrument presents the correct techniques of donning and doffing PPE, as well as the technique and appropriate materials for cleaning and disinfecting the ambulance.

At the end of each transportation, it is necessary to clean and disinfect all internal surfaces of the vehicle. Disinfection with 70% alcohol, sodium hypochlorite, or other disinfectant is indicated for this purpose, following the standard operating procedure defined for the cleaning and disinfection activity of the vehicle and its equipment^(20)^. In addition, hand hygiene with water and liquid soap or alcoholic hand preparation after cleaning the vehicle and removing the PPE^(8)^. The emergency units should have an area to execute terminal and concurrent ambulance cleaning before they return to the base, and vehicle doors and windows should be kept open during internal cleaning^(8,20)^. For those procedures, it is necessary to apply correct donning and doffing PPE, which should be removed after the process and during the work shift^(3,10,14)^.

In a study in which a checklist was created for the disinfection of ambulances transporting patients with COVID-19, the authors concluded that the use of a tool for cleaning and disinfecting the mobile unit contributes to reducing the chain of infection and provides security for professionals facing the pandemic^(8,20)^.

### Study limitations

The evaluation of the algorithm was performed by higher-level professionals, which can be considered a limitation of this study since the evaluation of the instrument by nursing technicians and assistants can produce different results.

### Contributions to the fields of Nursing, Health, or Public Service

The algorithm developed and validated in this study contributes to innovation in the work of nurses, physicians, physical therapists, and professionals in patient transport services. It assists mainly in decision-making about transporting patients with contagious infectious diseases, with guidelines for cleaning and disinfection of the ambulance and donning and doffing PPE. The information provided in the algorithm is important because if the techniques are not applied correctly, those professionals may become infected by the coronavirus and transmit it to other patients in their care. In addition, the instrument will provide subsidies to keep health professionals updated about the theoretical and practical approach to this content.

## CONCLUSIONS

The algorithm to guide health care professionals in cleaning and disinfecting the ambulance after caring for patients with contagious infectious diseases was developed and validated by nurses, physicians, and physical therapists who work on the frontline of the fight against contagious infectious diseases. The consensus was obtained among the judges in the second evaluation, indicating that this instrument can be applied as a training tool for professionals to help with minimal risk and safety without damage or adverse events.
